# Optimization of Polyphenol Extraction From Fresh Tea Shoots for Production of the Functional Cracker: Antioxidant Capacity and Bioaccessibility

**DOI:** 10.1002/fsn3.71652

**Published:** 2026-03-17

**Authors:** Dondu Unalan, Bige Karaman, Nihal Turkmen Erol, Ferda Sari

**Affiliations:** ^1^ Faculty of Agriculture, Department of Food Engineering Bursa Uludag University Bursa Turkey; ^2^ Department of Crop and Animal Production, Sivas Vocational School of Higher Education Sivas Cumhuriyet University Sivas Turkey

**Keywords:** bioactives, digestion, enrichment, HPLC, response surface methodology (RSM), tea shoots

## Abstract

In this study, the extraction conditions for fresh green tea shoots ( *Camellia sinensis* ) were optimized using Box–Behnken design (BBD) to maximize polyphenol yield in the green tea leaf extract (GTLE). The lyophilized GTLE obtained under optimal conditions was incorporated into cracker formulations, replacing a portion of the flour. The physicochemical properties of both the GTLE and the enriched crackers were evaluated, including antioxidant capacity (AC), total polyphenol (TP), and total flavonoid (TF) contents, bioaccessibility, individual phenolic compounds (via high performance liquid chromatography (HPLC), texture, and color values). BBD optimization revealed that a solid‐to‐solvent (S/S) ratio of 1:50.64, extraction temperature of 95°C, and duration of 20 min were ideal for polyphenol extraction. Enriched crackers exhibited significantly higher TP and TF contents compared to control crackers, with an AC of 12.24 mmol ascorbic acid equivalent (AAE)/g dry matter (DM), whereas no antioxidant activity was detected in the control. Bioaccessibility of polyphenols decreased during both gastric and intestinal digestion stages. Phenolic compounds, such as gallic acid (GA), epigallocatechin (EGC), epigallocatechin gallate (EGCG), quercetin‐3‐rhamnosylglucoside (Q3RG) and kaempferol‐3‐rhamnosylglucoside (K3RG), were identified in enriched crackers but absent in the control. The incorporation of GTLE also resulted in a decrease in lightness (L*) and enhancement in redness (a*) and yellowness (b*) of the crackers. These findings demonstrate that GTLE enrichment enhances the functional properties of crackers, particularly their antioxidant profile, making them a promising functional food product.

## Graphical abstract

Extraction of fresh tea polyphenols and functional cracker production stages.

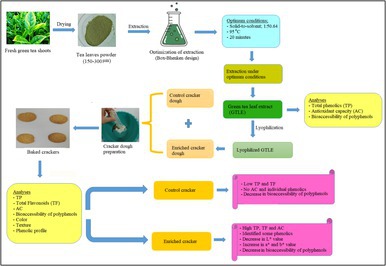

## 1 Introduction

Tea leaves are among the plants that contain high amounts of polyphenols, one of the bioactive compounds, and thus have many positive effects on health. Numerous studies on this subject have revealed that tea polyphenols (mainly catechins) have many beneficial effects such as antioxidant, antimutagenic, anticarcinogenic, and antibacterial activity (Kowalska et al. 2021; Wang et al. 2020; Teng et al. 2021). Regular consumption of tea polyphenols has also been associated with improved cardiovascular protection, glycemic control, immune modulation, and reduction of age‐related degenerative processes (Tang, Meng, Gan, et al. 2019). Recently, commercial tea extracts, especially obtained from fresh tea leaves, have been used as food antioxidants, nutritional supplements, cosmetic additives, and even medicine in addition to the direct consumption of tea. Therefore, achieving maximum recovery and activity of bioactive compounds requires careful optimization of extraction conditions (such as solvent type, concentration, temperature, and method). Otherwise, inappropriate processing may degrade phenolic compounds or lead to low yields (Zhang et al. 2019). Studies on the use of tea leaf for enrichment of foods have shown that it made a significant contribution to the sensorial, nutritional, or microbiological quality of the product. Recently, there has been increased focus on natural antioxidants than artificial ones because they are more economical and do not cause toxicity problems (Kazemi et al. 2017). One of these fortified products is cracker, which is a widely consumed bakery product. When white flour is used as raw material in the production of bakery products including crackers, the nutritive content of the final product is considerably reduced. Because important nutritional elements are removed during the milling of wheat. In recent years, studies on the enrichment of these products have become increasingly important in order to improve their quality and functionality (Sharma and Zhou 2011; Yu et al. 2023). For example, fortification of crackers with alternative flours (cereals, legumes, and agricultural by‐products) significantly improved total phenolic content, antioxidant activity, and digestibility while maintaining acceptable sensory quality (Chatziharalambous et al. 2023). However, in the literature, no study was found on the use of tea shoots extract, which is very rich in polyphenols, to add functionality to cracker.

The purposes of this research were (1) to optimize the extraction conditions through RSM and BBD to extract the most TP from shade‐dried fresh tea shoots (2) to produce enriched cracker by adding GTLE to the cracker formulation (3) to determine the AC, TP, and TF amounts, in vitro bioaccessibility of bioactives and individual phenolic contents of dried leaf and cracker samples by HPLC and additionally (4) to assess the crackers in terms of color and texture.

## 2 Materials and Methods

### 2.1 Materials

In the present study, the fresh tea shoots harvested from a tea garden in Rize, Turkey, were used. The shoots were dried at room temperature for 36 h without waiting and stored in polyethylene bags at 4°C prior to use. Flour, salt, sunflower oil, and eggs used for cracker production were supplied from the local market. All chemicals and reagents employed in the study were of HPLC or analytical grade.

### 2.2 Experimental Design for RSM Modeling

The equations of second‐order polynomials were applied to fit the experimental data in this research. The parameters (S/S ratio, temperature and time) for extraction of tea shoot polyphenols were optimized by use of BBD and RSM. The selection of independent variables (solid/solvent ratio, temperature and time) was done on the basis of preliminary studies and previous studies. The experimental design was shown in Table 1.

**TABLE 1:** Experimental and predicted values for TP and AC of tea shoots extracts in BBD.

Exp.	Independent variables			Dependent variable			
				TP (mg GAE/g DM)		AC (g AAE/100 g DM)	
	A	B	C	Experimental	Predicted	Experimental	Predicted
1	−1	−1	0	57.28	58.49	8.14	8.66
2	0	0	0	66.11	66.35	14.65	14.60
3	−1	0	1	70.47	67.83	12.37	11.85
4	1	1	0	83.76	82.69	17.00	16.41
5	−1	1	0	60.60	60.98	11.29	11.91
6	0	0	0	64.45	66.35	14.04	14.60
7	0	−1	−1	68.70	67.51	10.16	10.44
8	0	1	−1	72.47	72.75	14.81	16.03
9	0	1	−1	74.20	72.75	16.69	16.03
10	1	0	−1	71.94	72.85	13.41	13.52
11	1	−1	0	62.50	68.53	9.37	10.66
12	0	0	0	66.70	66.35	14.80	14.60
13	1	−1	0	75.38	68.53	12.55	10.66
14	0	1	1	85.43	86.69	16.38	16.52
15	−1	0	−1	59.34	59.36	11.94	11.25
16	0	0	0	68.62	66.35	14.88	14.60
17	1	0	1	86.47	86.10	15.83	16.07
18	−1	0	1	68.64	67.83	11.61	11.85
19	0	−1	−1	68.96	67.51	10.41	10.44
20	1	1	0	83.91	82.69	16.68	16.41
21	0	−1	1	75.13	75.29	13.09	13.11
22	1	0	1	86.08	86.10	15.15	16.07
23	0	0	0	66.11	66.35	14.43	14.60
24	−1	1	0	60.55	60.98	11.94	11.91
25	1	0	−1	70.31	72.85	13.35	13.52
26	−1	−1	0	57.42	58.49	8.32	8.66
27	0	0	0	66.12	66.35	14.78	14.60
28	−1	0	−1	59.03	59.36	11.73	11.25
29	0	−1	1	74.31	75.29	13.70	13.11
30	0	1	1	85.32	86.69	16.96	16.52
Independent variables					Levels		
					−1	0	+1
A	Solid/solvent (w/v)				1/25	1/50	1/75
B	Temperature (°C)				85	90	95
C	Time (min)				10	15	20

Analysis of variance (ANOVA) was used to assess the impact of each factor and evaluate the predicted model for the response variables (TP and AC). Data analysis was performed using MINITAB 17 software (State College, PA).

### 2.3 Extraction of Polyphenols

The samples of tea shoots (85.2% DM) and cracker defatted with petrol ether were ground and extracted in a falcon tube with water at a known temperature (B, %) in a hot water bath. Extraction was carried out at a known S/S ratio (A, w/v) defined in Table 1. After extraction time (*C*, min), the suspension was rapidly cooled, centrifuged, and filtered. The clear extract was stocked at −18°C till analyses. The applied conditions for extraction are demonstrated in Table 1.

### 2.4 Lyophilization of the Extract

The leaf extract obtained under the optimized conditions was frozen at −80°C and then dried in a lyophilizer under −50°C and 0.1 mbar (0.75 mmHg) vacuum for 21 h to be used in cracker preparation. Lyophilisates were stocked in brown tubes at −18°C until used.

### 2.5 Cracker Production

Cracker production was carried out according to the recipe applied by İncedayı and Türkmen Erol (2022). The freeze‐dried leaf extract was used as a flour substitute, at a rate of 1% of the dough's weight. Cracker without the extract constituted the control group.

### 2.6 Physical Analyses

The color and texture analyses were performed using the colorimeter (Konica‐Minolta CM 3600d, USA) and the Texture Analyzer TA.XT 2Plus (Stable Micro Systems Ltd., Surrey, UK).

### 2.7 Chemical Analyses

TP of the crackers was measured using the Folin–Ciocalteu reagent following the ISO 14502‐1:^2005^ method. AC was measured using the 2,2,diphenyl−1‐picryl‐hydrazyl (DPPH) method outlined by Türkmen Erol et al. (2009). TF and bioaccessibility were performed according to Rodrigues et al. (2016) and Minekus et al. (2014), respectively. For the phenolic profile of extracts, a previously published method (Chebbi et al. 2024) was employed using an HPLC system.

### 2.8 Statistical Analysis

Experimental results were assayed using SPSS software (SPSS statistics 23, IBM.2015) and presented as means ± standard deviation of triplicate measurements. One‐way ANOVA was performed, followed by the Duncan post hoc test. *p*‐value of < 0.05 was considered statistically significant.

## 3 Results and Discussion

### 3.1 RSM Modeling

The TP and AC values of the water extracts from the fresh tea shoots are demonstrated in Table 1. The values of TP and AC of the extracts ranged from 57.28 to 86.47 mg GAE/g DM and from 8.14 to 17.00 g AAE/100 g DM, respectively.

**FIGURE 1:**
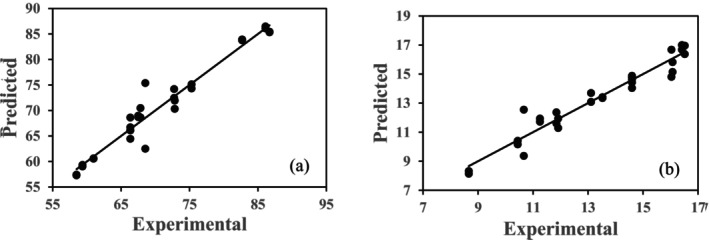
Experimental and predicted values for TP (a) and AC (b) of the extracts.

As seen in Table 2, the model was highly significant (*p* < 0.05) with high determination coefficients (*R* ^2^), indicating a strong correlation between the experimental and predicted data for TP and AC values of the extracts (Figure 1).

**TABLE 2:** ANOVA of RSM modeling for TP and AC of the extracts.

	Source	DF^a^	SS^b^	MS^c^	*F* value	*p*
TP	Model	9	2236.92	248.55	40.36	0.000
	Linear	3	1757.71	585.90	95.14	0.000
	Solid/solvent	1	1008.76	1008.76	163.80	0.000
	Temperature	1	276.99	276.99	44.98	0.000
	Time	1	471.96	471.96	76.64	0.000
	Square	3	380.74	126.91	20.61	0.000
	S/S*S/S	1	13.54	13.54	2.20	0.154
	Temperature*Temperature	1	52.88	52.88	8.59	0.008
	Time*Time	1	315.43	315.43	51.22	0.000
	Interaction	3	98.47	32.82	5.33	0.007
	S/S*Temperature	1	68.12	68.12	11.06	0.003
	S/S*Time	1	11.43	11.43	1.86	0.188
	Temperature*Time	1	18.92	18.92	3.07	0.095
	Residual Error	20	123.17	6.16		
	Lack‐of‐Fit	3	26.15	8.72	1.53	0.244
	Pure error	17	97.02	5.71		
	Total	29	2360.09			
	*R* ^2^ = 94.78 Adj‐*R* ^2^ = 92.43 Pred‐*R* ^2^ = 87.07					
AC	Model	9	170.373	18.9304	33.01	0.000
	Linear	3	133.194	44.3979	77.42	0.000
	Solid/solvent	1	42.267	42.267	73.70	0.000
	Temperature	1	81.016	81.0158	141.27	0.000
	Time	1	9.910	9.9103	17.28	0.000
	Square	3	29.771	9.9236	17.30	0.000
	S/S*S/S	1	23.098	23.098	40.28	0.000
	Temperature*Temperature	1	6.205	6.205	10.82	0.004
	Time*Time	1	0.875	0.875	1.53	0.231
	Interaction	3	7.409	2.4696	4.31	0.017
	S/S*Temperature	1	3.119	3.1188	5.44	0.030
	S/S*Time	1	1.907	1.9066	3.32	0.083
	Temperature*Time	1	2.383	2.383	4.16	0.055
	Residual Error	20	11.469	0.5735		
	Lack‐of‐Fit	3	2.955	0.9851	1.97	0.157
	Pure error	17	8.514	0.5008		
	Total	29	181.843			
	*R* ^2^ = 93.69 Adj‐*R* ^2^ = 90.85 Pred‐*R* ^2^ = 84.15					

^a^ Degree of freedom.

^b^ Sum of square.

^c^ Mean square.

### 3.2 Effect of Extraction Parameters on TP and AC of the Extracts

The S/S ratio, temperature and extraction time had significant linear effects on TP and AC (*p <* 0.05) as indicated in Table 2. The square terms of temperature and extraction time except S/S ratio had significant impacts on TP (*p <* 0.05). But the square terms of S/S ratio and temperature except extraction time had significant effects on AC (*p <* 0.05). Furthermore, for both responses, the interaction between the S/S ratio and temperature was significant (*p <* 0.05). The equations used to fit the experimental data were given below:

(1) $$\mathrm{TP}=66.35+7.940A+4.161B+5.431C-1.354A^{2}+2.676B^{2}+6.536C^{2}+2.918\mathrm{AB}+1.195\mathrm{AC}+1.538\mathrm{BC}$$

(2) $$\mathrm{AC}=14.597+1.625A+2.250B+0.787C-1.769A^{2}-0.917B^{2}+0.344C^{2}+0.624\mathrm{AB}+0.488\mathrm{AC}-0.546\mathrm{BC}$$

According to Equations (1) and (2), TP and AC increased with increasing S/S ratio, temperature, and extraction time owing to positive coefficients of these variables.

**FIGURE 2:**
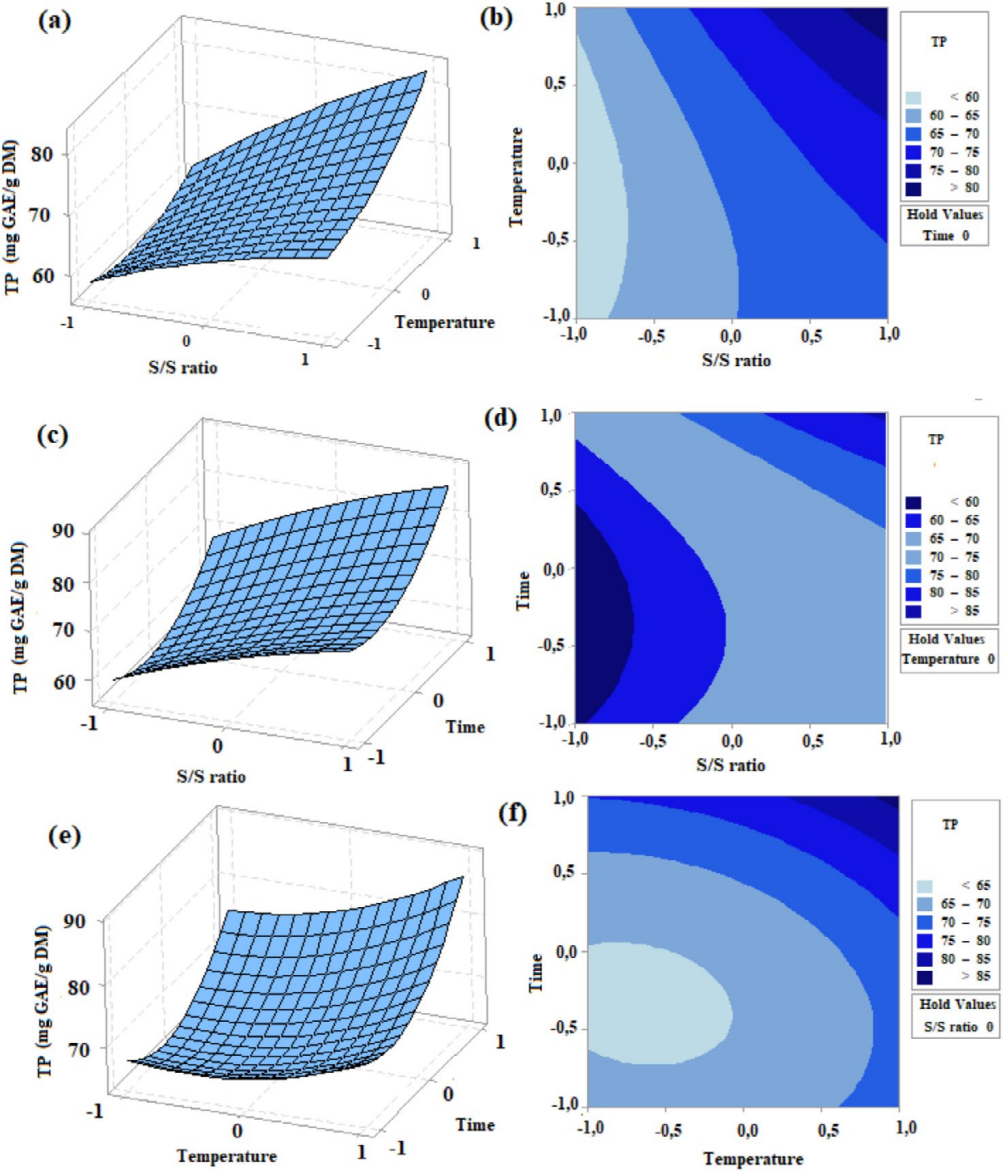
Response surface plots (a, c, and e) and contour plots (b, d, and f) indicating the effects of independent variables on TP from tea shoots. The extraction time was maintained constant at 15 min (a, b); temperature was kept at 90°C (c, d), and S/S ratio was kept constant at 1/50 of ratio (e, f).

**FIGURE 3:**
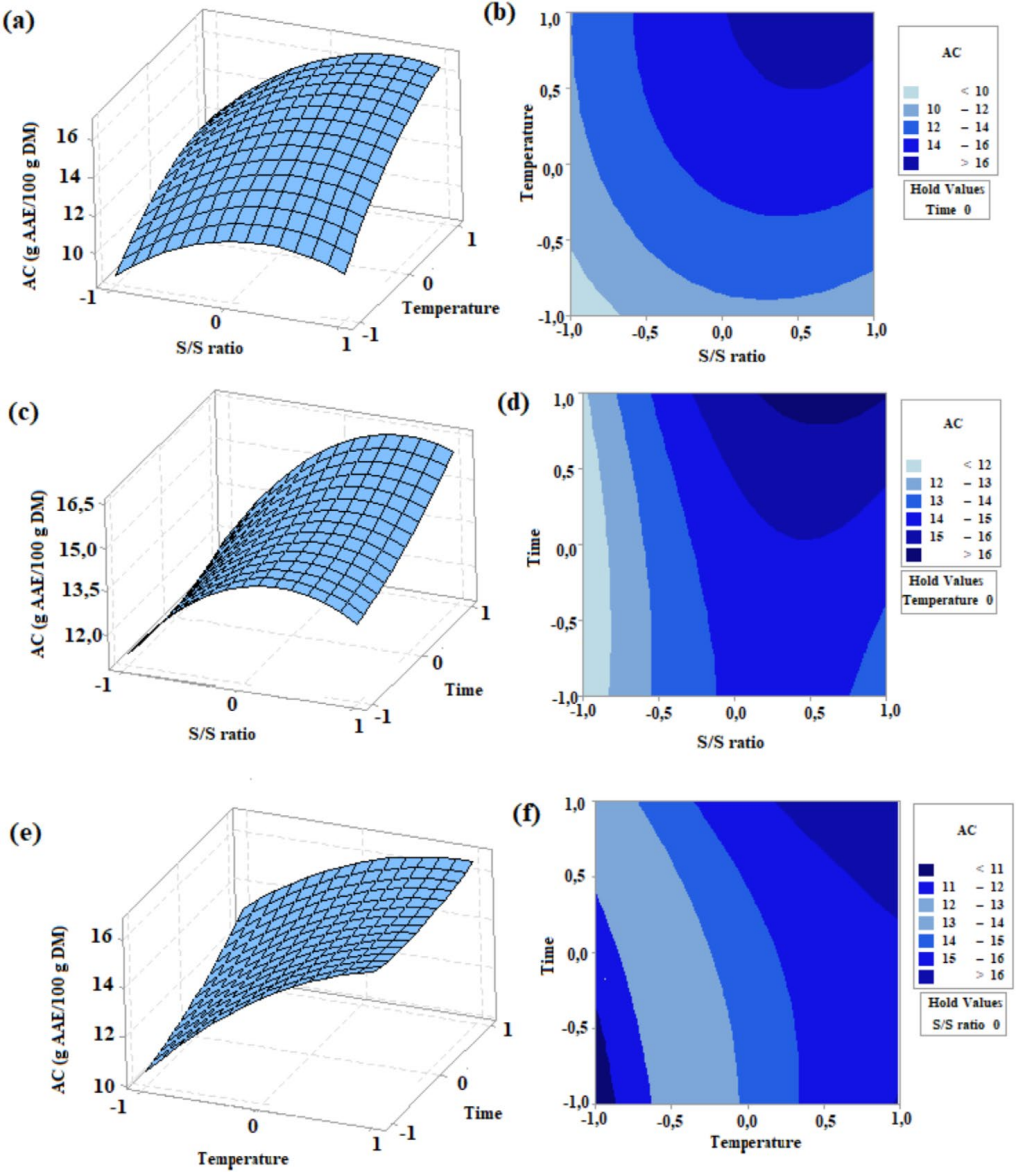
Response surface plots (a, c, and e) and contour plots (b, d, and f) indicating the effects of independent variables on AC from tea shoots. Extraction time was maintained constant at 15 min (a, b); temperature was kept at 90°C (c, d) and the S/S ratio was kept constant at 1/50 of ratio (e, f).

Figures 2 and 3 display three‐dimensional response surface and contour plots as a function of S/S ratio, temperature, and time. As illustrated in Figures 2a–f and 3a–f, temperature from 85°C (code = −1) to 95°C (code = +1) caused high TP and AC. This can be attributed to the fact that high temperatures soften tissues and increase solubility (Rebollo‐Hernanz et al. 2021). As an agreement with our result, TP contents of cacao shell (Rebollo‐Hernanz et al. 2021) and pitaya seeds (Shi et al. 2022) increased when the temperature increased.

Among the extraction parameters, the S/S ratio had a significant impact on TP (*p* < 0.05). As shown in Figure 2a–d, increasing the S/S ratio from 1/25 (code = −1) to 1/75 (code = +1) resulted in a higher TP value.

When the S/S ratio increases, the contact area between the target compound and the solvent increases, which allows more phenolic compounds to be extracted (Shi et al. 2022). Regarding AC, S/S ratio from 1/25 (code = −1) to 1/50 (code = 0) yielded high AC. Nevertheless, a decreasing trend towards 1/75 (code = +1) was observed. In consistency with this result, in the study of Zhou et al. (2019), antioxidant activity increased as the S/S ratio increased from 1/10 to 1/25 and reached the maximum level between 1/25 and 1/40. The ratio of 1/29.3 was also determined as the optimal point.

### 3.3 Optimization Conditions

The optimal factor levels were identified through optimization using the Box–Behnken design to achieve the highest TP value from fresh tea leaves. The optimization process determined the ideal conditions as a S/S ratio of 1/50.64, an extraction temperature of 95°C, and an extraction time of 20 min. Under these conditions, the predicted TP value was 97.39 mg GAE/g DM. The model's validity was then assessed, and the experimentally measured amount was 99.07 mg GAE/g DM, showing that the predicted value closely aligned with the experimental outcome at the optimal conditions. While many studies have focused on optimizing polyphenol extraction from various sources, comparing the optimal extraction conditions from this study with those found in earlier limited research (Andres et al. 2020; Fadjare Frempong et al. 2021) is challenging due to variations in extraction methods and materials used, despite the use of similar independent variables.

### 3.4 TP, AC, and In Vitro Digestion of Tea Extract

TP value (99.07 mg GAE/g DM) obtained from this study (Table 3) was in consistent with those reported by Roshanak et al. (2016) (109.85 mg GAE/g DM for fresh tea leaves dried in the shade) and Erturk et al. (2010) (33.00–291.18 mg GAE/g for fresh tea leaves dried in air at 45°C). Differing from these results, Somsong et al. (2020) determined the TP value of freeze dried tea leaves as 379.01 mg GAE/g DM. This difference may be attributed to variations in the extraction method, cultivars, climatic conditions, harvesting season, and geographic locations. On the other hand, the TP results determined in this research were also compared to those of green tea that is recognized for its high polyphenol content. Accordingly, in certain earlier studies (Donlao and Ogawa 2018; Tang, Meng, Gan, et al. 2019), TP values of green tea were reported as 66.91–173 mg GAE/g DM. This indicated that fresh tea leaves could be a more cost‐effective source of polyphenols, as they are unprocessed in contrast to green tea. AC value (1104.55 mmol AAE/g DM) of the extract was determined by DPPH method. Few studies in the literature have focused on the antioxidant activity of fresh tea leaves. Additionally, these studies employed various modifications of the DPPH method, used different standards, and reported results in different units. As a result, a direct comparison of the present findings with literature values was not feasible.

**TABLE 3:** TP (mg GAE/g DM) and AC (mmol AAE/g DM) values of the extract and bioaccessibility (%) of TP.

	Initial	Gastric stage	Intestinal stage
TP	99.07 ± 0.28^c^	53.26 ± 1.71^b^	45.62 ± 1.42^a^ *
AC	1104.55 ± 14.75^c^	574.11 ± 29.36^b^	223.69 ± 20.06^a^
TP bioaccessibility	100.00 ± 0.00^c^	53.76 ± 1.66^b^	46.05 ± 1.33^a^

* The differences between the means, indicated by lowercase letters in the same row, are significant (*p* < 0.05).

The TP and AC values of the extract followed a closer pattern after digestion, both showing a significant decrease (*p* < 0.05) in comparison to their initial values (Table 3). The most notable reductions were seen during the intestinal phase, which is linked to the reduced stability of polyphenols in the alkaline environment of intestinal digestion (Fawole and Opara 2016). As far as we know, no studies have been published on the changes in polyphenols and AC of fresh tea leaves following digestion. However, when compared to studies on the alteration of green tea polyphenols, Rodrigues Silva et al. (2021) and Shu et al. (2019) reported the bioaccessibility of green tea polyphenols as 25.9% and 9.69%–15.57%, respectively, both less than the result found in this study (53.76%). Additionally, Green et al. (2007) found a decrease of about 80.4% in total catechins after gastric and intestinal digestion, with the most significant reduction occurring during the intestinal stage. The reductions of TP and thus AC after gastrointestinal digestion have also been shown in the previous studies (Fawole and Opara 2016; Wang et al. 2017) with different foods.

### 3.5 TP, TF, AC, and In Vitro Digestion of Crackers

Effect of tea extract on TP, TF, AC and bioaccessibility of polyphenols of cracker after digestion was presented in Table 4. The results demonstrated that the enrichment with the extract enhanced the TP and TF amounts and AC of the enriched cracker compared to the control one which had a lower amount of TP and TF and no AC. Consistent with our findings, Radočaj et al. (2014) also reported that the addition of green tea leaves increased TP content and antioxidant activity of cracker. Similarly, fortification with green tea polyphenols of mango powder caused higher TP and antioxidant activity compared to the unfortified product (Tan et al. 2023). The rise in the AC of the enriched cracker could be linked to the higher TP content due to the addition of tea extract.

**TABLE 4:** TP (mg GAE/g DM), AC (mmol AAE/g DM), TF (mg RE/g DM), and bioaccessibility (%) of polyphenols and flavonoids of crackers enriched with tea extract.

	Cracker type	Initial phase	Gastric phase	Intestinal phase
TP	Control	0.80 ± 0.01^b^	0.82 ± 0.017^b^	0.75 ± 0.009^a^ *
	Enriched cracker	2.53 ± 0.03^c^	1.71 ± 0.06^b^	1.39 ± 0.09^a^
TP bioaccessibility	Control	100.00 ± 0.00^b^	102.92 ± 2.36^b^	93.81 ± 2.19^a^
	Enriched cracker	100.00 ± 0.00^c^	67.59 ± 3.06^b^	54.94 ± 2.97^a^
AC	Control	nd	nd	nd
	Enriched cracker	12.24 ± 1.06^b^	10.92 ± 0.66^b^	3.47 ± 0.15^a^
TF	Control	2.20 ± 0.16	nd	nd
	Enriched cracker	3.62 ± 0.36^b^	1.11 ± 0.08^a^	nd
TF bioaccessibility	Control	100.00 ± 0.00	nd	nd
	Enriched cracker	100.00 ± 0.00^b^	30.66 ± 1.72^a^	nd

* The differences between means, indicated by lowercase letters in the same row, are statistically significant (*p* < 0.05).

In vitro digestion had a significant impact on the TP, TF and AC of the crackers (*p* < 0.05) (Table 4). The same trend for TP of enriched cracker was observed as in the case of the extract. After digestion, TP value of the cracker significantly reduced relative to its initial amount (*p* < 0.05). This could be attributed to physicochemical changes in the gastrointestinal tract (temperature, pH and enzymes) (Donlao and Ogawa 2018; Rodrigues Silva et al. 2021), as well as the interaction of polyphenols with other dietary components released during digestive process (Debelo et al. 2020). The reduction of TP amount after digestion has also been determined by He et al. (2016) for fruit juices and Chen et al. (2013) for tea juices. Otherwise, while TP content of control sample aligned with its initial content after gastric stage (*p* > 0.05), it significantly decreased after intestinal stage (*p* < 0.05). As shown in Table 4, bioaccessibility of polyphenols in the enriched cracker significantly (*p* < 0.05) reduced after digestion and also were less than that of the control one. Although protein determination was not included within the scope of this study, it is estimated that the protein amount of enriched cracker containing less flour is lower than control cracker. For this reason, the lower bioaccessibility of enriched cracker is could be due to (1) the insufficient amount of proteins to bind polyphenols in this sample as polyphenols are shielded by binding to proteins and (2) the changes in their individual phenolic profiles, thus variations in their stabilities. Previous studies reported that polyphenol–protein complexes improved the stability of polyphenols in the gastrointestinal environment during in vitro digestion conditions, leading to greater bioaccessibility of polyphenols (Ahmadi et al. 2023; Niu et al. 2024; Mao et al. 2024). After digestion, control cracker had no AC as in the beginning. However, AC of enriched cracker significantly (*p* < 0.05) decreased in accordance with the decrease in the amount of TP. In line with our findings, a reduction in antioxidant activity after in vitro digestion was reported by Lucas‐González et al. (2021) for enriched spaghetti with persimmon flour and grape pomace (S. Wang et al. 2017). After the digestion process, for both crackers TF was not detected. However, bioaccessibility of flavonoids in enriched cracker after gastric stage was 30.66% unlike control one (Table 4). A reduction in TF following in vitro digestion has also been reported by other authors who have addressed this issue in quinoa (Balakrishnan and Schneider 2020) and pasta (Kasprzak‐Drozd et al. 2024).

### 3.6 Phenolic Profile of Crackers

**FIGURE 4:**
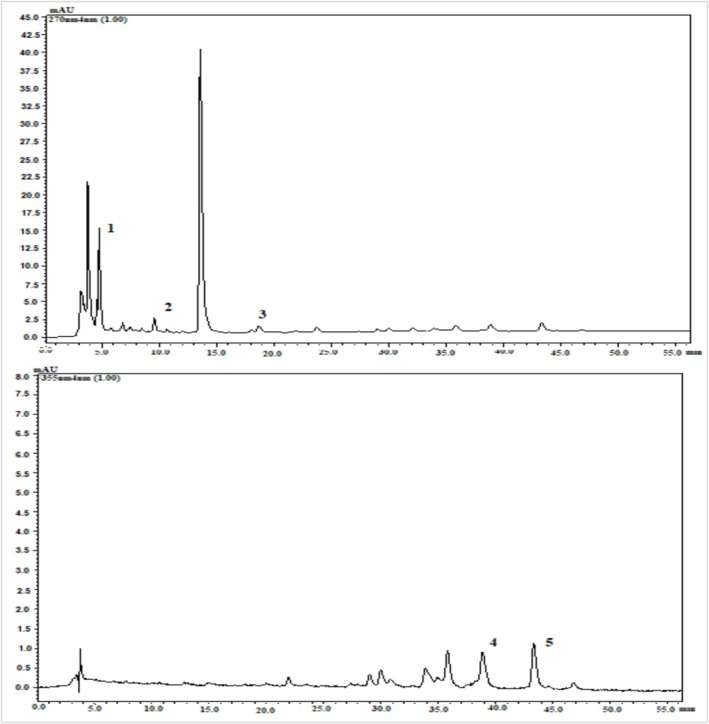
HPLC chromatogram of extract of GTLE enriched cracker, where 1‐GA, 2‐EGC, 3‐EGCG, 4‐Q3RG, 5‐K3RG.

Phenolic compounds of the samples were examined by HPLC and as a result of the analysis, GA, EGC, EGCG, Q3RG, and K3RG were identified and quantified in enriched cracker (Table 5 and Figure 4). However, none of the phenolic compounds investigated was found in the control cracker. Similarly, in the study of Sedej et al. (2011) while rutin and quercetin were detected in the cracker produced using buckwheat flour, no phenolic compounds were found in the control sample. As seen in Table 5, K3RG from flavonols was the most abundant phenolic compound, followed by GA, which was the only phenolic acid detected. The breakdown of gallated catechins during cracker production due to their instability may increase the amount of gallic acid in the cracker. Because catechins are prone to epimerization and degradation (Sharma and Zhou 2011). The stability of tea catechins can be primarily influenced by factors such as pH, temperature, oxygen concentration, free radicals, metal ions, and enzymes (Wang et al. 2008; Ahmad et al. 2023). Regarding flavanols, the amount of EGC was found to be higher than EGCG; although in tea leaf extract, EGCG was higher than EGC according to our previous study (Chebbi et al. 2024) and several researches in the literature (Lee et al. 2014; Liu et al. 2020; Luo et al. 2025). This may be due to the difference in their stability during cracker production (Sharma and Zhou 2011).

**TABLE 5:** Phenolic profile of cracker with GTLE.

Phenolic compound	Amount (mg/100 g DM)
GA	10.30 ± 0.22
EGC	6.48 ± 0.57
EGCG	3.80 ± 0.25
Q3RG	9.24 ± 0.12
K3RG	23.29 ± 0.27

### 3.7 Color and Texture of Cracker

Color and texture results belong to crackers are given in Table 6. The L* value varies from 0 (black) to 100 (white) was lower in enriched cracker when compared to the control one. This result could be attributed to the increase in its polyphenol content. The reduction in the L* value promotes the increase in the darkness of the sample. In agreement with our result, in the study of Ou et al. (2018) where cookies were enriched with different polyphenols (resveratrol, epicatechin and rosmarinic acid) in different levels, lower polyphenol addition levels did not impact color development, while higher levels led to increased browning. Similar result was also reported by Venkatachalam and Nagarajan (2017). Otherwise, a* and b* values of enriched cracker increased as seen in Table 6. This was similar with the result reported by Altunkaya et al. (2013) for bread enriched with pomegranate peel powder.

**TABLE 6:** Texture and color values of crackers.

	Control cracker	Cracker with GTLE
L*	80.69 ± 0.07	67.97 ± 0.48
a*	2.99 ± 0.06	6.68 ± 0.25
b*	23.61 ± 0.08	26.73 ± 0.23
Hardness (g.s.)	837.89 ± 7.97	945.02 ± 60.82

Hardness of the enriched cracker was higher compared with the control. The increase in hardness value could be caused by the reduced moisture. Because moisture of control cracker (14.88%) was higher than that of enriched one (12.10%) (data not shown). Similar result was observed for cracker with germinated lentil extract (Polat et al. 2020) and noodles enhanced by various tea products (Han et al. 2020).

## 4 Conclusion

The polyphenol extraction from shade‐dried tea leaves was optimized using RSM, and the optimum extraction conditions were successfully identified in this study. The analysis revealed that the S/S ratio, extraction temperature, and extraction time had a significant linear impact on TP and AC (*p* < 0.05). GTLE was freeze‐dried and incorporated into a cracker formulation. The enriched crackers demonstrated significantly higher levels of TP and TF against control crackers, which exhibited no detectable AC. Among the phenolic compounds analyzed in the enriched cracker, K3RG was the most abundant, followed by GA. Both GTLE and the enriched crackers experienced significant reductions in TP and AC following in vitro digestion, particularly after the intestinal digestion stage (*p* < 0.05). These findings highlight the potential of GTLE as a natural, clean‐label ingredient for the bakery sector. Its incorporation into a cracker not only enhances AC but also provides a functional alternative to conventional formulations typically low in phenolic compounds. This research therefore offers promising insights for the food industry regarding the development of functional bakery products with added health benefits.

## Author Contributions

**Dondu Unalan:** investigation, formal analysis, validation. **Bige Karaman:** data curation, resources, supervision, writing – review and editing. **Nihal Turkmen Erol:** supervision, conceptualization, methodology, writing – original draft. **Ferda Sari:** formal analysis, methodology, validation, visualization. All authors reviewed the manuscript.

## Funding

This work was supported by TÜBİTAK.

## Ethics Statement

The authors have nothing to report.

## Consent

The authors have nothing to report.

## Conflicts of Interest

The authors declare no conflicts of interest.

## Data Availability

The data that support the findings of this study are available from the corresponding author upon reasonable request.
